# Maxillary posterior intrusion with corticotomy-assisted approaches with zygomatic anchorage—a finite element stress analysis

**DOI:** 10.1186/s40510-019-0262-4

**Published:** 2019-03-04

**Authors:** Cemile Uysal, Burcu Baloş Tuncer, Cumhur Tuncer

**Affiliations:** 1 Private Practice, Istanbul, Turkey; 2 Gazi University, Faculty of Dentistry, Department of Orthodontics, 8 Cadde, 06510 Ankara, Turkey

**Keywords:** Posterior maxillary intrusion, Zygomatic anchorage, Corticotomy, Finite element stress analysis

## Abstract

**Background:**

Anterior open bite is one of the most difficult malocclusions to treat and maintain in orthodontics. An effective treatment approach to correct anterior open bite is the intrusion of maxillary posterior teeth. The aim of this study was to evaluate the effects of corticotomy-assisted posterior maxillary intrusion with zygomatic anchorage by using finite element stress analysis.

**Methods:**

An acrylic bite block on the posterior teeth including two transpalatal arches were modeled and 1.96 N intrusive force was loaded. Three scenarios were set, first with no subapical corticotomy, second with buccal, and third with both buccal and palatal corticotomies. The stress distributions along the cortical, cancellous bone surfaces, and dental structures were assessed by finite element stress analysis.

**Results:**

Stress distributions over cortical and cancellous bones were commonly located at the inferior curvature of the zygomatic buttress area and posterior teeth for all scenarios. Stress values above the apices of anchor teeth were decreased for both corticotomy scenarios. Increased stress distributions were observed in cancellous bone around corticotomy regions. Despite the acrylic appliance and transpalatal arches, the stresses along the posterior teeth were not uniform. The apical third of the first molar mesiobuccal apex showed the highest stress values in all scenarios.

**Conclusions:**

Corticotomy-assistance effected biomechanical responses of dentoalveolar structures during maxillary posterior intrusion. There was no apparent difference for the stress levels of the root apices between corticotomy scenarios, pointing out that only buccal corticotomy may be a better option in corticotomy-assisted maxillary intrusion.

## Background

Several treatment alternatives have been proposed for anterior open bite correction depending on the severity of the skeletal discrepancies and/or dental compensations [1–4]. Maintaining the increased vertical dimensions, correction of the posterior dentoalveolar structures and steep mandibular plane are critical during treatment. Main problems that clinicians might encounter during anterior open bite treatment include concerns for facial esthetics, unwanted dentoalveolar changes, and risks of relapse.

Over-erupted maxillary posterior dentition has been addressed as one of the main morphological characteristics of this malocclusion [5], and the challenges of posterior tooth intrusion treatment have been highlighted as anchorage problems, undesirable tooth movements, and/or root resorption [6]. Numerous studies have evaluated posterior intrusion mechanics with the aid of skeletal anchorage devices as an alternative to conventional mechanics [7–9], and they have reported true molar intrusion, reduced anterior facial height, and mandibular plane angle [8]. Alternatives for maxillary posterior anchorage have been pointed out by De Clerck et al. [10], Erverdi et al. [11], and Sherwood et al. [8]. Erverdi et al. [9] investigated the effectiveness of maxillary posterior teeth intrusion by using titanium mini-plates placed at the zygomatic area, and they have stated that the zygomatic area was a useful anchorage site for this treatment.

Despite the improvements in treatment mechanics, intrusion can increase the risk of root resorption due to the resistance at the interradicular area [12]. In 2013, Li et al. [13] reported a volumetric measurement of root resorption after molar intrusion with mini-screw, and observed a prominent volume loss especially in the mesiobuccal root of the upper first molar teeth. Besides, intrusion of posterior teeth may be challenging especially in adults, due to the lack of growth compensation, histological changes of the supporting tissues, reduced blood supply, and as mentioned, due to the increased risk of root resorption [14]. In this respect, the introduction of corticotomy-assisted orthodontics provided new solutions to some limitations in orthodontic tooth movement by a localized area of increased bone turnover through a controlled surgical damage [15–17]. The controlled surgical injury causes a temporary and reversible decrease in regional bone density, which reduces the resistance to tooth movement, thus enabling rapid tooth movement at the early stages [18]. The additional advantages of corticotomy-assisted orthodontics has been described as increased treatment efficiency, less need for extraoral appliances, less relapse [19], and less risk of root resorption [20]. However, corticotomy-assisted orthodontics is an invasive surgical procedure, which requires the elevation of buccal and often a palatal/lingual flap [21]. Consequently, a better understanding of the mechanics of intrusion could lead to a more appropriate selection of treatment approaches, appliance designs, and more efficient treatments. Due to the challenges during anterior open bite treatments, there is a need to further analyze the most effective treatment options and the need for analyzing buccal and/or palatal corticotomy assistance. This knowledge would be beneficial both for the patients and the clinicians to achieve the least invasive approach with the most effective treatment results.

Biomechanics is important in orthodontic treatments, since stress fields occur in the supporting tissues when a force is applied. Finite element analysis can be used to simulate different orthodontic treatment approaches as an effective and non-invasive method [22]. A recent study has performed a finite element analysis to evaluate the stress and strains with maxillary posterior intrusion mechanics [23]. However, knowledge regarding the biomechanical effects of intrusion by the assistance of corticotomy is limited. Therefore, the aim of this study was to evaluate the mechanical impacts of corticotomy-assisted posterior maxillary intrusion with zygomatic anchorage on dentoalveolar structures by using finite element stress analysis. Three scenarios were established for this purpose; the first including no corticotomy, the second including a buccal subapical corticotomy, and the third consisting of both buccal and palatal subapical corticotomies.

## Materials and methods

This study was performed with the approval of Gazi University Institute of Health Sciences (22/12/2014-E.131593). The computed tomography (CT) images of a previously conducted adult maxillary bone with a skeletal class 1 adult patient without any craniofacial anomalies [24] were acquired at 0.2 mm intervals in the axial direction perpendicular to the occlusal plane. Three-dimensional (3D) model of the skull and the maxillary bone was constructed by 3D Doctor software. 3D finite element models were constructed and generated by using Algor Fempro software (Algor, Inc. 150 Beta Drive Pittsburgh, PA 15238-2932, USA), VRMesh Studio (Virtual Grid Inc., Bellevue City, WA, USA) analysis programmes, and Rhinoceros 4.0 software (Rhinoceros Inc., Seattle, USA).

Maxillary teeth models were set according to the prescription by Wheeler [25], and constructed with 3D smart optics scanner (Activity 880, Smart Optics, Sensor Technik GmbH, Bochum, Germany). Maxillary dental arch was arranged in medium Tru-arch form (Ormco, Orange, CA, USA), and the inclination and angulation of each tooth were set according to the prescription by Roth [26]. The alveolar bone was built up at 1 mm away from and along the curvature of the cemento-enamel junction. The periodontal ligament thickness was uniformly set at 0.25 mm, in accordance with previous studies [23, 27]. The computer-aided design model of Leibinger Universal mini-plates (Stryker Corporation, Germany), and self-drilling mini-screws with 1.7 mm diameter/6 mm length were generated with 3D smart optics scanner.

### Appliance design

**Fig. 1:**
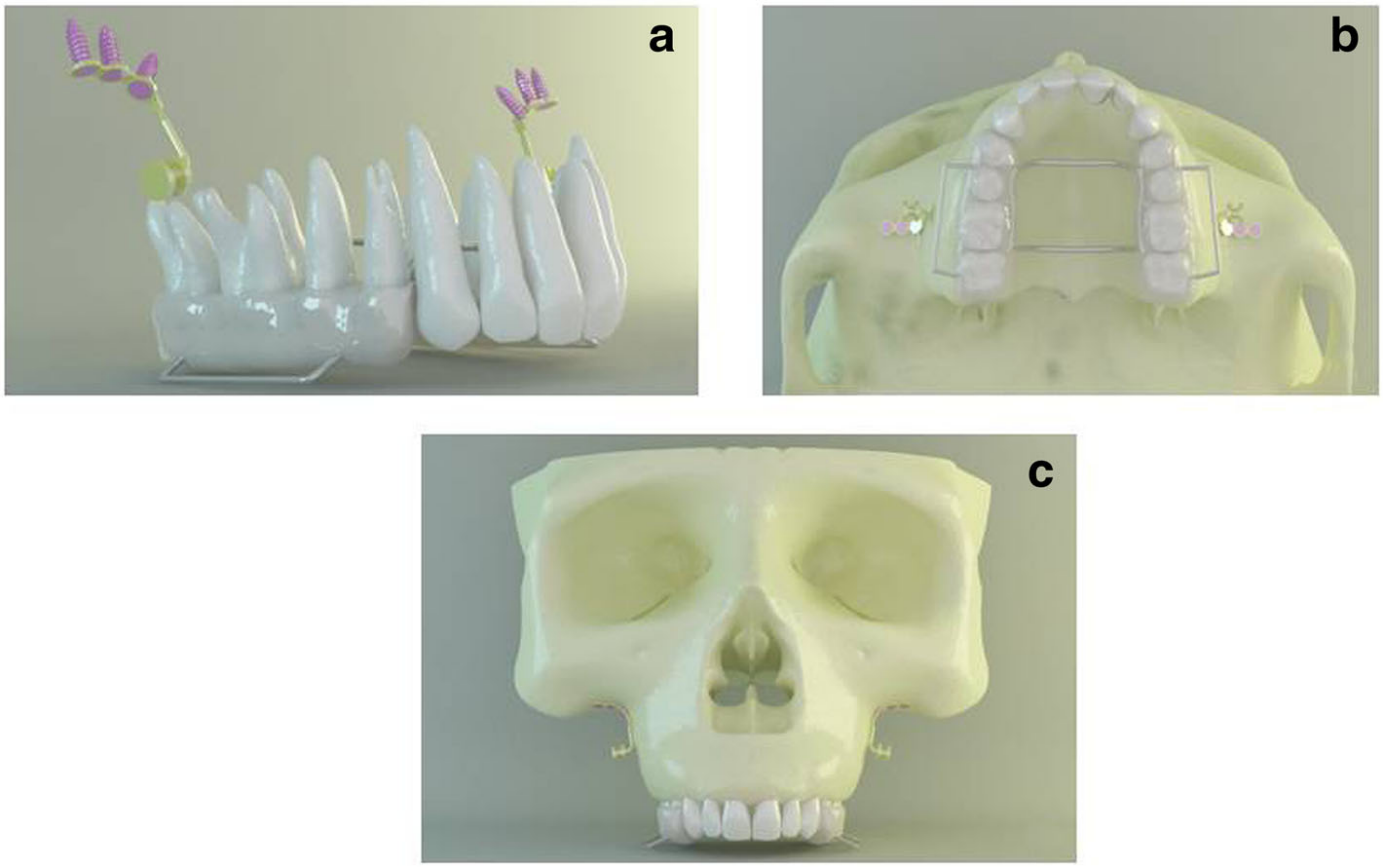
**a** View of the acrylic appliance and the anchorage unit. **b** Occlusal view of the appliance. **c** First scenario with no corticotomy

An acrylic appliance on the maxillary posterior teeth (premolars and molars) connected with two palatal arches (1.4 mm diameter round stainless steel) was constructed in accordance with the study of Erverdi et al. [4]. In accordance with the clinical applications, transpalatal arches were adapted evenly 5 mm from the palatal bone to achieve clearance for the intrusion movement. A 0.9 mm round stainless steel wire was attached on the buccal side, to apply the intrusive force to the anchorage unit, and the wire has been adjusted so that the intrusive force could be applied parallel to the long axis of the first molars. The anchorage unit contained the mini-plates, which were mounted on the zygomatic buttress area between the first and second maxillary molar roots and three mini-screws (1.7 mm diameter, 6 mm length) which were constructed to fix the mini-plates (Fig. 1a, b). Transpalatal arch-acrylic interfaces and the vestibular side connectors were defined as fully bonded surfaces to simulate a rigid connection.

**Fig. 2:**
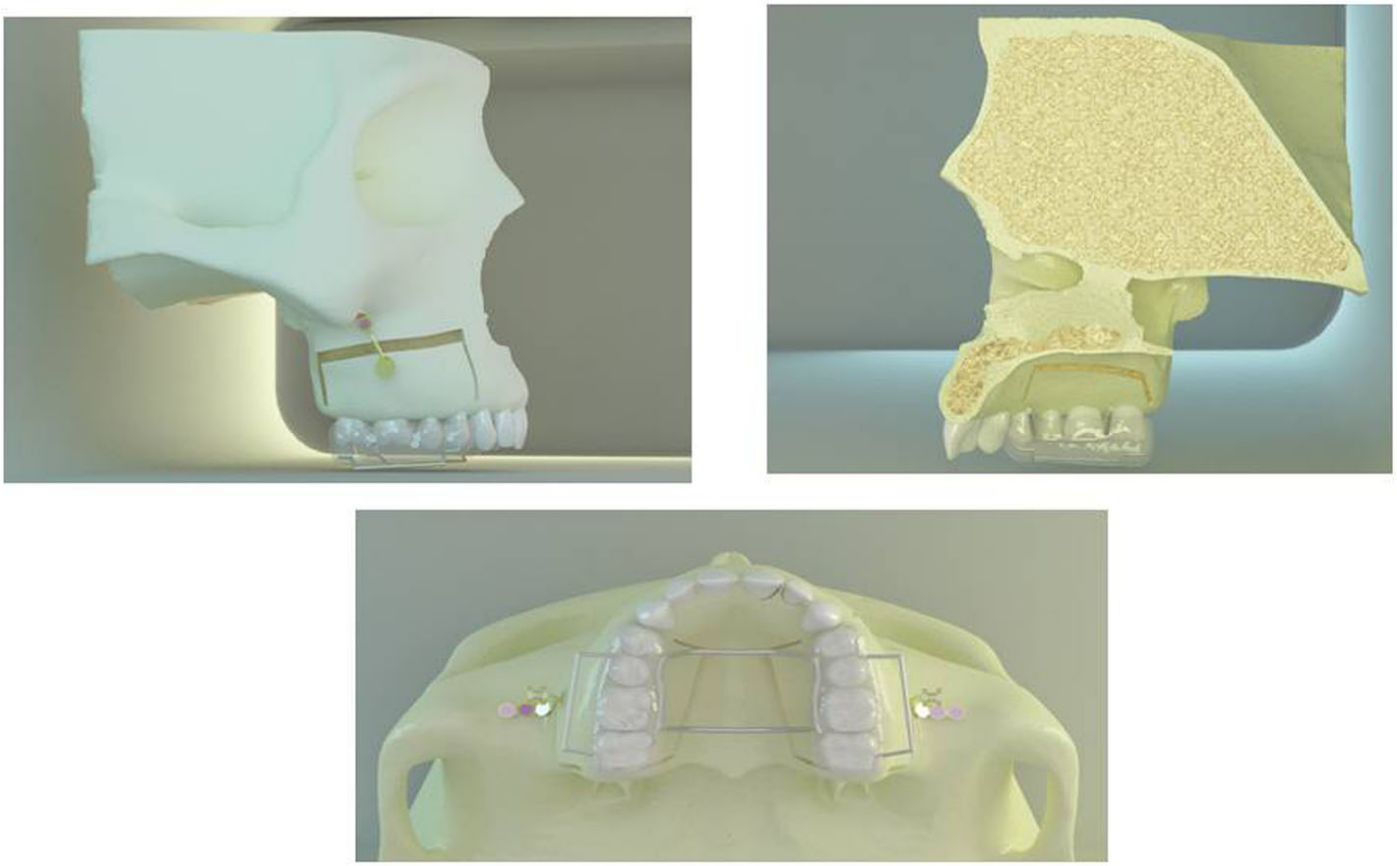
Models presenting buccal and palatal subapical corticotomies

Three scenarios were set. The first scenario presented application of intrusive force from the anchorage unit to the acrylic appliance with no corticotomy (Fig. 1c). The second scenario included intrusion assisted by buccal subapical corticotomy. For this purpose, vertical incisions were performed 2–3 mm above the interdental alveolar margin till 5–6 mm above the apices at the distal side of maxillary canines and distal side of second molars with a gap of 1–1.5 mm diameter. A horizontal incision was performed 5 mm above the apices of posterior teeth with a 3-mm diameter (Fig. 2). Third scenario contained intrusion with buccal and palatal subapical corticotomies, which has been presented previously by Akay et al. [28]. Vertical and horizontal incisions at the buccal side were designed as in the second scenario. In this scenario, a palatal corticotomy was carried out through the palatal curvature, performed from the distal side of maxillary canines toward distal side of second molars (Fig. 2). The incisions were limited at the cortical bone and did not involve the cancellous bone.

The final solid meshes were configured as a tetrahedron, with eight nodes. In scenario 1, 567,870 elements and 124,800 nodes; in scenario 2, 551,189 elements and 120,074 nodes; and in scenario 3, 557,884 elements and 122,486 nodes were used. Mechanical properties of materials in the models were obtained from experimental data in previous reports [29–31] (Table 1), and all structures were assumed to be isotropic, homogeneous, and linearly elastic. The superior and posterior skull regions formed the boundaries of the model, as zero displacement in the *x*, *y*, and *z* directions (transverse, anteroposterior, and vertical axes, respectively). The magnitude of intrusive force for all models was defined as 1.96 N (200 g) [4, 32].

**Table 1:** Mechanical properties of the materials in the model

Variable	Elastic modulus (GPa)	Poisson ratio
Compact bone	15	0.30
Cancellous bone	1.5	0.30
Teeth	1.86	0.31
Stainless steel	200	0.29
Mini-plate and mini-screw (titanium)	110	0.35
Acrylic block (polymethyl methacrylate)	0.015	0.35

*GPa* gigapascal

By using finite element stress analysis, the Von Mises stress distribution along the cortical and cancellous bone surfaces, and dental structures were evaluated. The estimated stress values were given in megapascals (MPa). The stress regions on the teeth and the anchorage unit were also evaluated. The stress distribution was presented by color contour bands, where different colors declared different stress levels.

## Results

The stressed areas in the skull model presented highest stress at the inferior curvature of the zygomatic buttress region, which was followed by the posterior teeth for all scenarios.

### Stress distributions over cortical bone

The stress values over the cortical bone for all scenarios are shown in Table 2. The simulations tested in this study showed that Von Mises stresses were commonly located at the inferior curvature of the zygomatic buttress area for all models, and presented the highest stress with 55 × 10^−3^ MPa.

**Table 2:** Von Mises stress values over the cortical bone

	Scenario 1 (MPa)	Scenario 2 (MPa)	Scenario 3 (MPa)
Anterior nasal spine	9 × 10^−3^	13 × 10^−3^	14 × 10^−3^
Infraorbital foramen	12 × 10^−3^	14 × 10^−3^	13 × 10^−3^
Zygomatic buttress	55 × 10^−3^	55 × 10^−3^	55 × 10^−3^
Superior of buccal horizontal corticotomy	30 × 10^−3^	7 × 10^−3^	6 × 10^−3^
Inferior of buccal horizontal corticotomy	25 × 10^−3^	20 × 10^−3^	21 × 10^−3^
Maxillary tuberosity	8 × 10^−3^	39 × 10^−3^	45 × 10^−3^
Palatal alveolar process	29 × 10^−3^	39 × 10^−3^	25 × 10^−3^
Palatine process	11 × 10^−3^	12 × 10^−3^	4 × 10^−3^
İncisive foramen	23 × 10^−3^	30 × 10^−3^	32 × 10^−3^

Scenario 1, no corticotomy; scenario 2, buccal corticotomy; scenario 3, buccal and palatal corticotomy; *MPa* megapascal

**Fig. 3:**
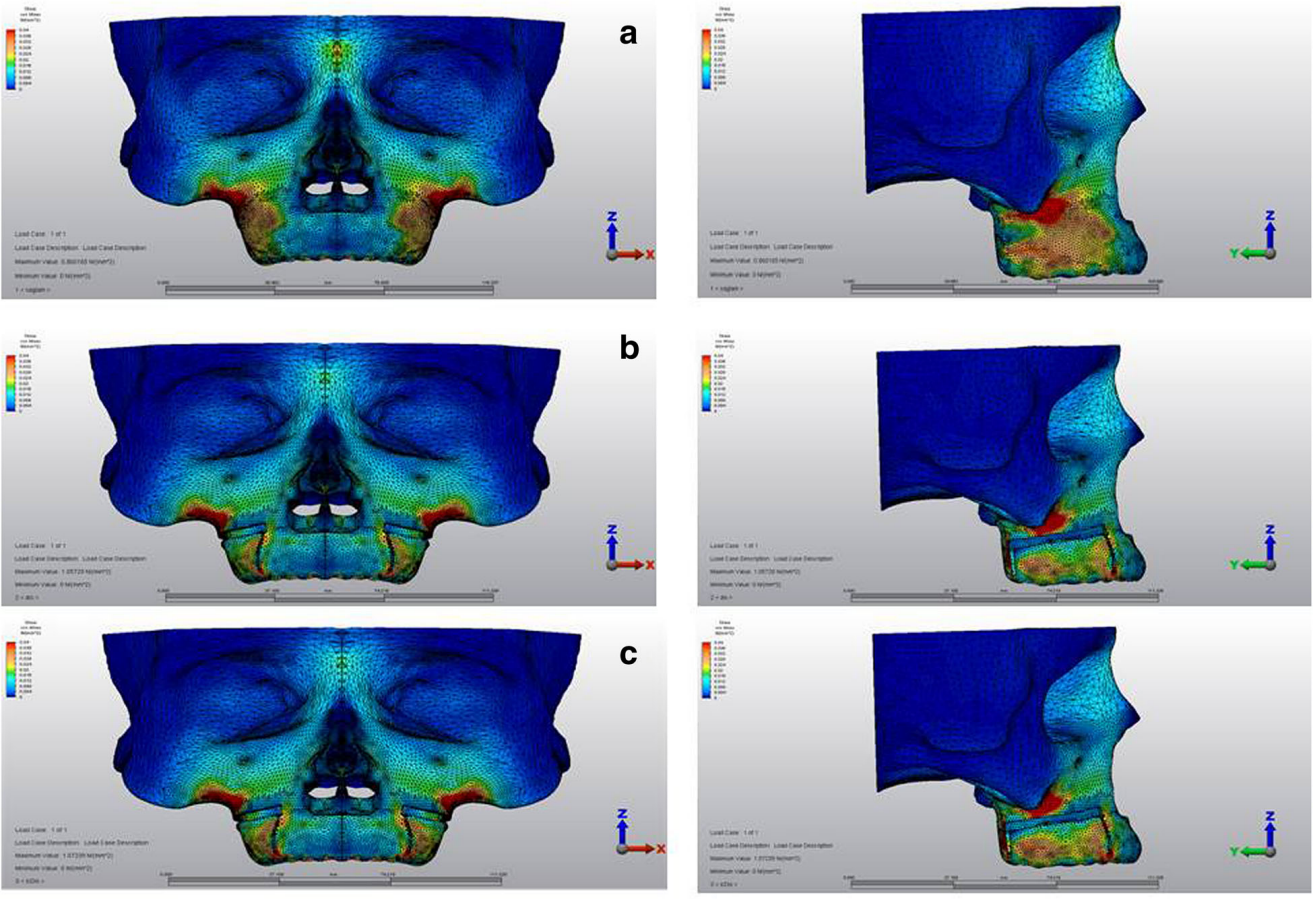
Stress distributions over cortical bone **a** first, **b** second, and **c** third scenarios

Stress values at the area above the apices of the anchor teeth in the first scenario was 30 × 10^−3^ MPa, which decreased for both of the corticotomy scenarios (7 × 10^−3^ MPa, 6 × 10^−3^ MPa, respectively). The second scenario with buccal corticotomy resulted in higher stress values for tuber maxilla with a calculation of 39 × 10^−3^ MPa when compared to the first scenario (8 × 10^−3^ MPa). Similarly, in the third scenario, stress was high for tuber maxilla (45 × 10^−3^ MPa) (Fig. 3).

**Fig. 4:**
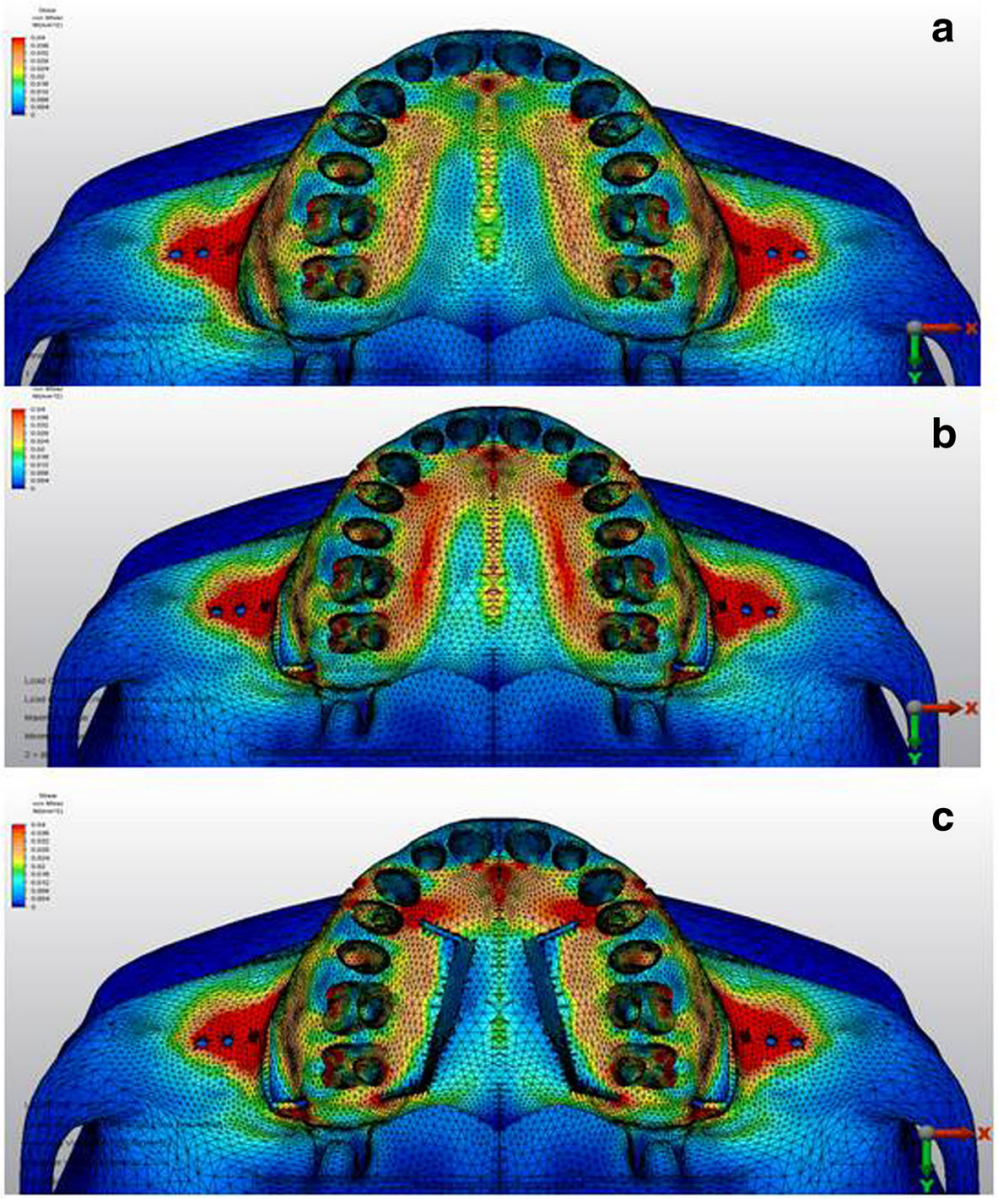
Stress distributions from occlusal view over cortical bone **a** first, **b** second, and **c** third scenarios

From the occlusal view, the first scenario showed that the palatal alveolar process of the anchor teeth demonstrated high stress with a value of 29 × 10^−3^ MPa, which was higher in the second scenario with a value of 39 × 10^−3^ MPa. In the third scenario, this stress was concentrated around the vertical incision between canine and first premolar, and the stress for the palatal alveolar process of the anchor teeth was lower than in other scenarios with a value of 25 × 10^−3^ MPa (Fig. 4).

### Stress distributions over cancellous bone

**Fig. 5:**
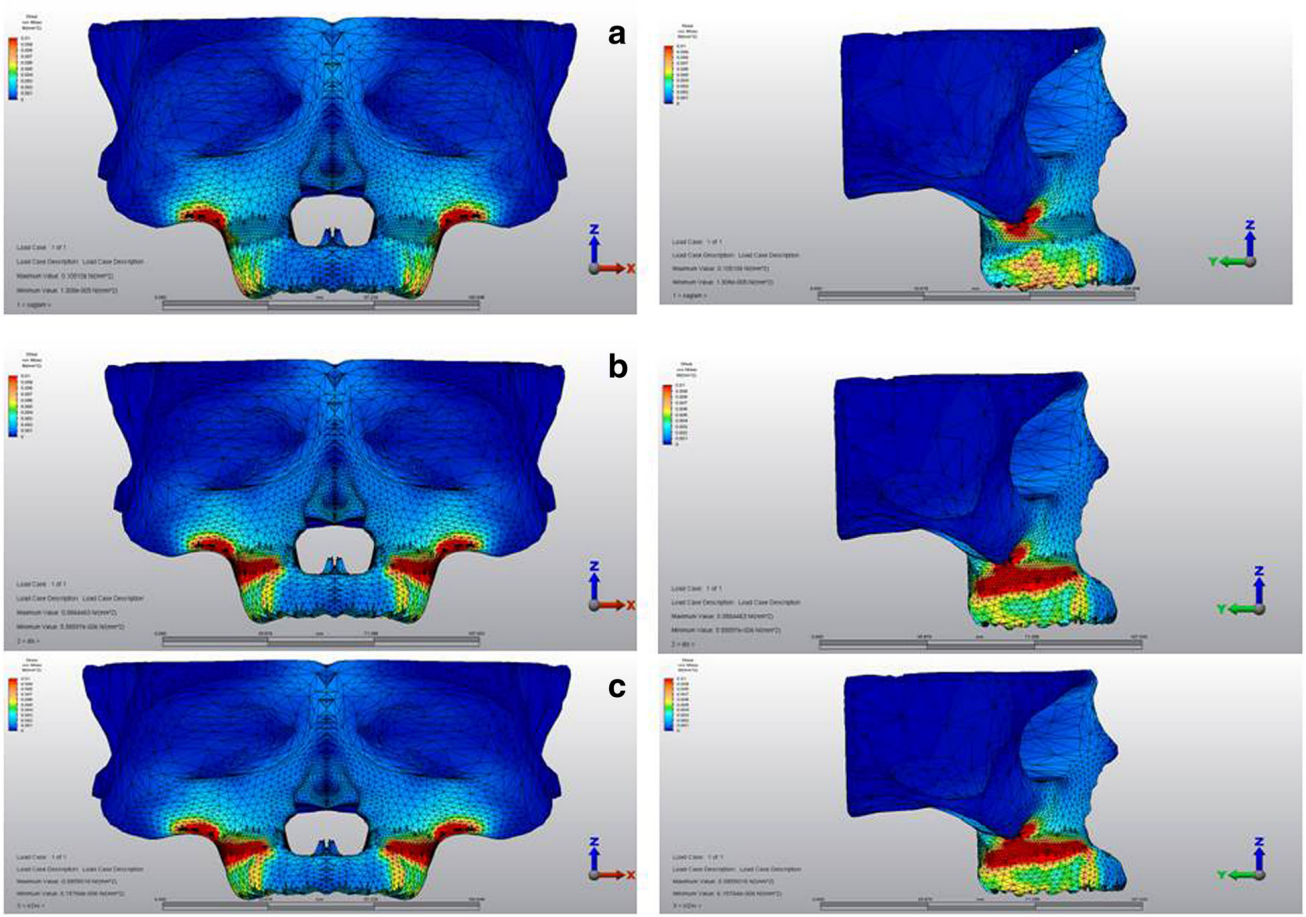
Stress distributions over cancellous bone **a** first, **b** second, and **c** third scenarios

The simulations revealed that stress values over the cancellous bone at the zygomatic buttress region were 20 × 10^−3^ MPa in all scenarios, as shown in Table 3 and Fig. 5.

**Table 3:** Von Mises stress values for the cancellous bone

	Scenario 1 (MPa)	Scenario 2 (MPa)	Scenario 3 (MPa)
Anterior nasal spine	1 × 10^−3^	1 × 10^−3^	1 × 10^−3^
Infraorbital foramen	1 × 10^−3^	1 × 10^−3^	1 × 10^−3^
Zygomatic buttress	20 × 10 ^3^	20 × 10^−3^	20 × 10^−3^
Buccal horizontal corticotomy	4 × 10^−3^	17 × 10^−3^	18 × 10^−3^
Buccal vertical corticotomy	1 × 10^−3^	5 × 10^−3^	6 × 10^−3^
Palatal horizontal corticotomy	2 × 10^−3^	2 × 10^−3^	14 × 10^−3^
Palatal vertical corticotomy	2 × 10^−3^	3 × 10^−3^	6 × 10^−3^

Scenario 1, no corticotomy; scenario 2, buccal corticotomy; scenario 3, buccal and palatal corticotomy; *MPa* megapascal

**Fig. 6:**
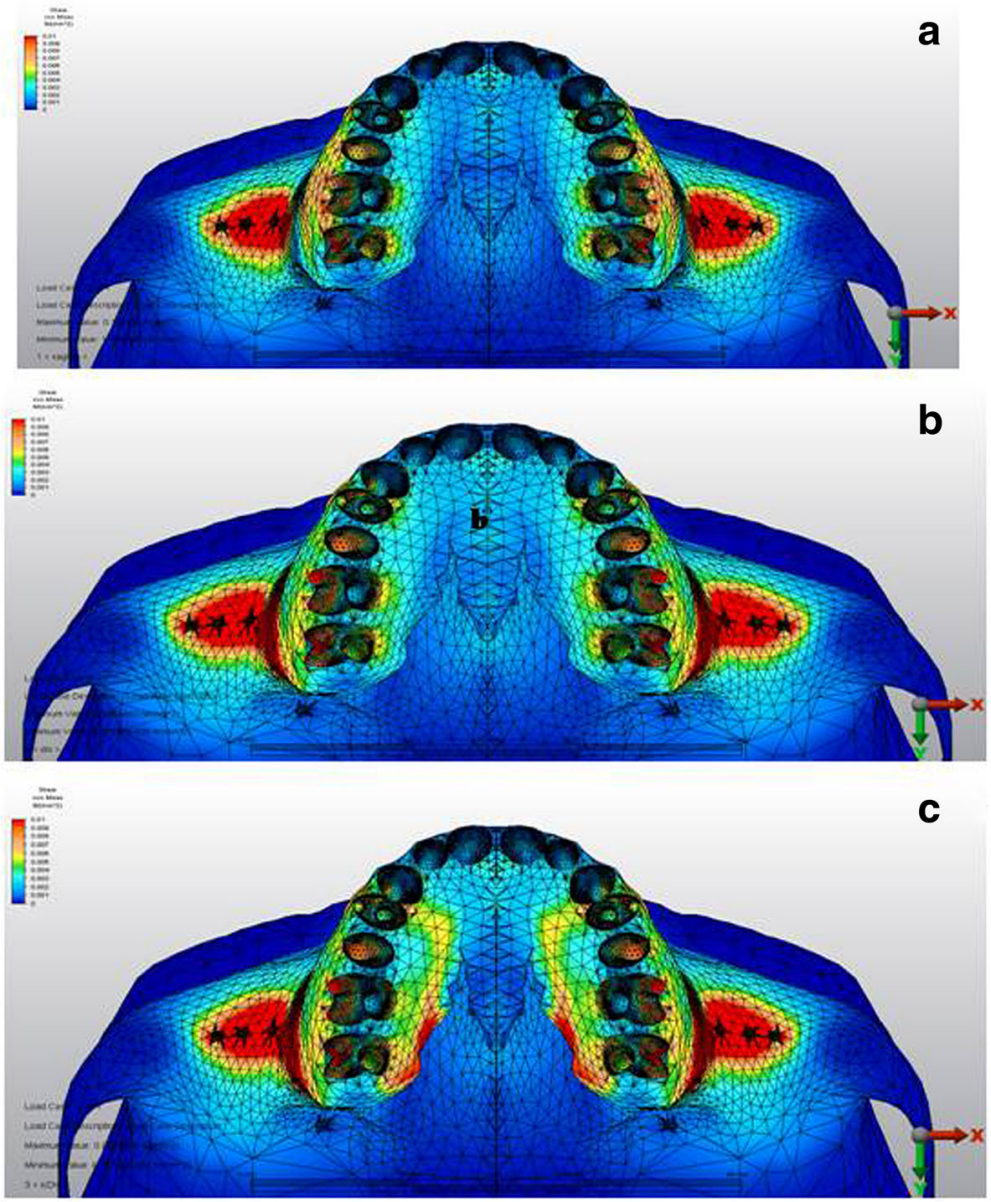
Stress distributions from occlusal view over cancellous bone **a** first, **b** second, and **c** third scenarios

In the first scenario, the stress value at the area above the apices of the anchor teeth was low (4 × 10^−3^ MPa). However, this value was higher in the corticotomy scenarios (17 × 10^−3^ MPa, 18 × 10^−3^ MPa, respectively) (Fig. 5). The stress value for the palatal horizontal and vertical incision regions showed higher values in the third scenario calculated as 14 × 10^−3^ MPa and 6 × 10^−3^ MPa, respectively (Fig. 6).

### Stress distributions over the anchorage unit and the intrusion appliance

**Fig. 7:**
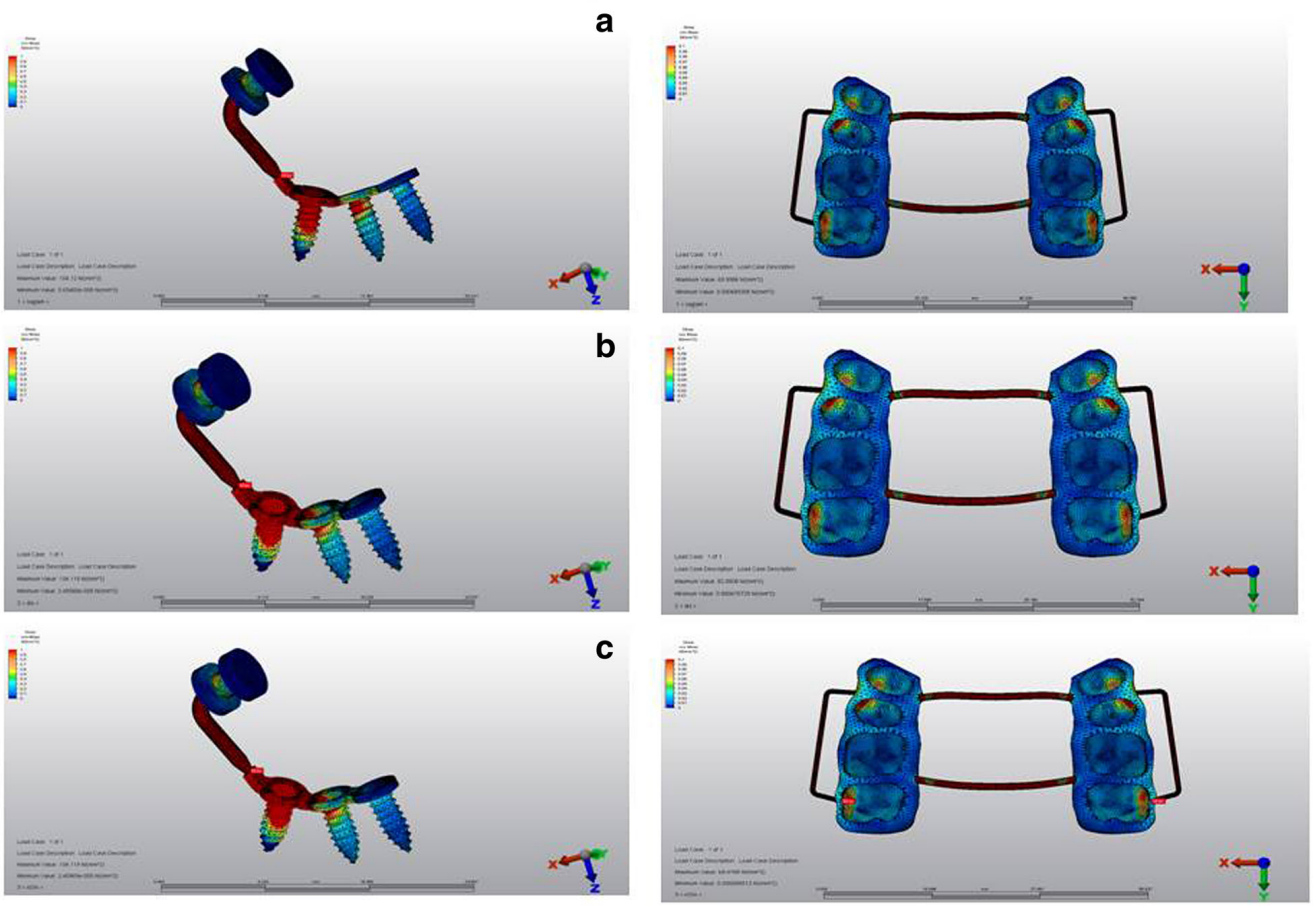
Stress distributions over anchorage unit and intrusion appliance **a** first, **b** second, and **c** third scenarios

All scenarios demonstrated high stresses at the connection area between the body and the intraoral region of the mini-plate. The highest stress was observed at the mini-screw, closer to the force application site. The buccal wire was exposed to high stress, followed by transpalatal arches for all scenarios. Compared to the wires, the stress at the acrylic appliance showed lower stress (Fig. 7).

### Stress distributions over the dental structures

**Fig. 8:**
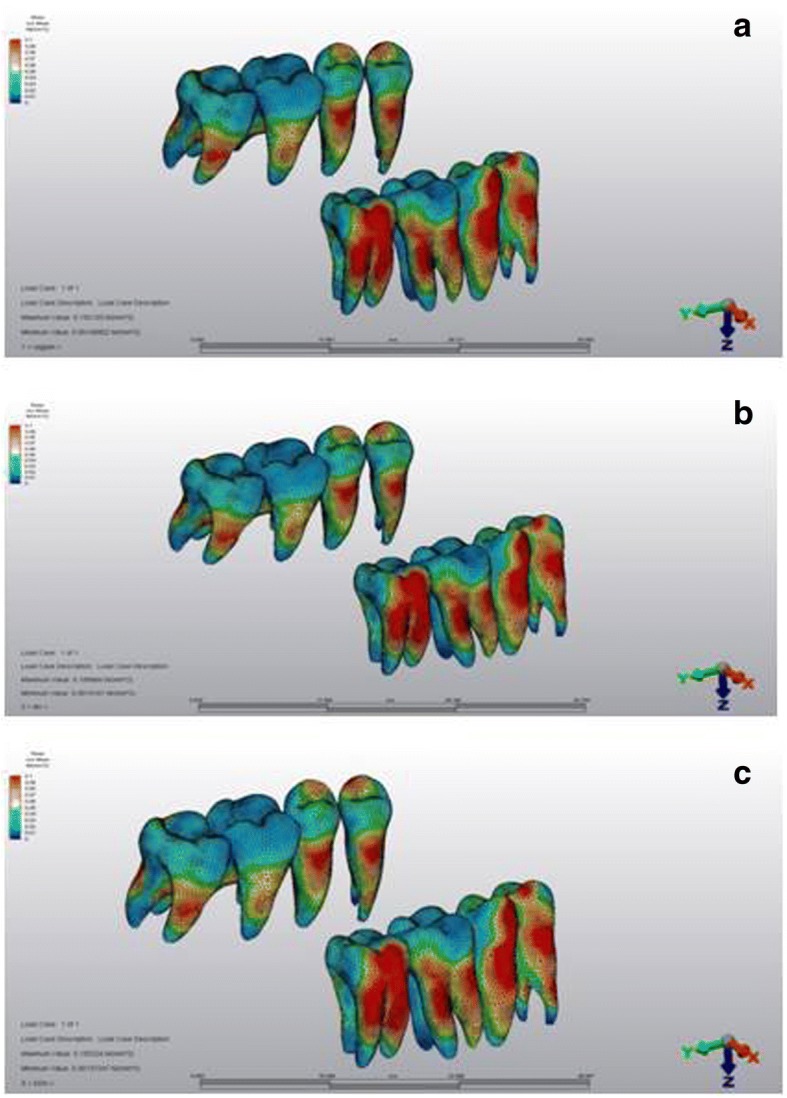
Stress distributions over the dental structures **a** first, **b** second, and **c** third scenarios

High stress values were recorded at the buccal surfaces of the premolars and second molars for all simulations (Fig. 8). Measurements at the cusp tips showed that stress distributions were not uniform at the occlusal surfaces of the teeth. The maximum Von Mises stresses were evident at the premolars’ buccal cusp tips, and the stress values were lower at the palatal cusps (Table 4).

**Table 4:** Von Mises stress values for the cusp tips

	Scenario 1 (MPa)	Scenario 2 (MPa)	Scenario 3 (MPa)
I. Premolar buccal	66 × 10^−3^	65 × 10^−3^	69 × 10^−3^
I. Premolar palatal	38 × 10^−3^	37 × 10^−3^	37 × 10^−3^
II. Premolar buccal	57 × 10^−3^	56 × 10^−3^	74 × 10^−3^
II. Premolar palatal	38 × 10^−3^	43 × 10^−3^	43 × 10^−3^
I. Molar mesiobuccal	21 × 10^−3^	21 × 10^−3^	21 × 10^−3^
I. Molar distobuccal	25 × 10^−3^	26 × 10^−3^	25 × 10^−3^
I. Molar palatal	24 × 10^−3^	22 × 10^−3^	21 × 10^−3^
II. Molar mesiobuccal	22 × 10^−3^	52 × 10^−3^	21 × 10^−3^
II. Molar distobuccal	52 × 10^−3^	43 × 10^−3^	48 × 10^−3^
II. Molar palatal	27 × 10^−3^	30 × 10^−3^	28 × 10^−3^

Scenario 1, no corticotomy; scenario 2, buccal corticotomy; scenario 3, buccal and palatal corticotomy; *MPa* megapascal

The measurements observed for the root apices showed that maximum Von Mises stresses were found at the apical third of the first molar mesiobuccal root for all scenarios, and the stresses were slightly increased for corticotomy scenarios (Table 5).

**Table 5:** Von Mises stress values for the root apices

	Scenario 1 (MPa)	Scenario 2 (MPa)	Scenario 3 (MPa)
I. Premolar buccal	1 × 10^−3^	2 × 10^−3^	1 × 10^−3^
I. Premolar palatal	3 × 10^−3^	2 × 10^−3^	3 × 10^−3^
II. Premolar	19 × 10^−3^	21 × 10^−3^	18 × 10^−3^
I. Molar mesiobuccal	45 × 10^−3^	51 × 10^−3^	52 × 10^−3^
I. Molar distobuccal	25 × 10^−3^	21 × 10^−3^	26 × 10^−3^
I. Molar palatal	11 × 10^−3^	8 × 10^−3^	11 × 10^−3^
II. Molar mesiobuccal	22 × 10^−3^	31 × 10^−3^	24 × 10^−3^
II. Molar distobuccal	25 × 10^−3^	32 × 10^−3^	36 × 10^−3^
II. Molar palatal	18 × 10^−3^	13 × 10^−3^	21 × 10^−3^

Scenario 1, no corticotomy; scenario 2, buccal corticotomy; scenario 3, buccal and palatal corticotomy; *MPa* megapascal

## Discussion

Orthodontic forces originate stress and strain on bone, and tooth movement is generated by the responses at the supporting tissues. Corticotomy has provide benefits for the clinicians in facilitating orthodontic tooth movement [33, 34], but the knowledge concerning the biomechanical effects are limited. In this study, a 3D finite element model was designed to simulate maxillary posterior intrusion assisted by different corticotomy approaches. A common result in all scenarios was the high stress levels for the inferior curvature of the zygomatic buttress area, and the buccal surfaces of the anchor teeth. Similarly, a recent study has pointed out the increased stress values adjacent to the force application sites [23].

In our study, the stress values along the cortical bone showed moderate distribution at the posterior buccal alveolar segment. For the corticotomy scenarios, transmission of the stress was interrupted by the horizontal corticotomy line, which might depend on the relief effect of the corticotomy cut. Chung et al. [34] have stated that corticotomy cuts can reduce the resistance of alveolar bone to orthodontic tooth movement by breaking the bone integrity. In line, a recent study showed that the stress dramatically declined during maxillary expansion above the corticotomy line, and they stated that the maxillary half splitted by the corticotomy cut could be expanded more easily when compared to the part with no corticotomy [24]. Our results also demonstrated that transmission of the stress at the palatal alveolar process of the anchor teeth was interrupted in the third scenario by the horizontal corticotomy line. Taken together, application of subapical corticotomies provided reduction of initial stresses 4–5 times at the buccal side, and 3 times at the palatal side. Similarly, a previous study reported the lowered stresses around anchor teeth during maxillary expansion with the combination of Le Fort and paramedian osteotomies [35]. Yang et al. [36] also interpreted their similar results as the reduction of the resistance to tooth movement by the assistance of corticotomies.

The current stress values for tuber maxillaries showed approximately five times higher values for the corticotomy scenarios. In this study, the vertical incisions were performed above 2–3 mm from the crest region in order to preserve bone integrity. Therefore, the stresses generated by the intrusive force were accumulated on the intact cortical bone margins, which might have contributed to this finding.

Another common finding was the stress regions at the cancellous bone, which were concentrated at the inferior curvature of the zygomatic buttress area. This finding might be related with the uninterrupted force transferred from the mini-plate and the mini-screws directly to the cancellous bone. Results also revealed that the corticotomy cuts multiplied the stresses for cancellous bone by three to seven times compared with the model with no corticotomy. This was in accordance with the results of Yang et al. [36], who had stated that the stress values were increased at the cancellous bone around corticotomy regions, and they declared that this might contribute to tooth movement by enhancing the biomechanical response. Corticotomy has been shown to facilitate orthodontic tooth movement by regional acceleration phenomenon, which results in healing of the injured bone by an increase in the rate of bone turnover [33]. The healing stimulus causes transient osteopenia in alveolar bone, leading to decreased resistance to tooth movement [18, 33]. The increased stress at the cancellous bone might show that the mechanical responses of dentoalveolar structures can be affected by corticotomy cuts during intrusion. Therefore, the effects of corticotomy have been reflected in the cancellous bone as a stimulating factor that can increase bone turnover, and the initial responses of posterior teeth toward orthodontic force. However, it would be beneficial to construct further studies to evaluate biological responses through bone remodeling activities in gingival crevicular fluid, or the rate of tooth movement.

When the stress distributions at the anchorage unit and the intrusion appliance were examined, all scenarios showed that the point of attachment of the mini-plate to the oral cavity, and the buccal wire of the appliance were exposed to high stresses, followed by the palatal arches. Clinically, these may reveal the need of preparing the intraoral appliances as rigid as possible.

With respect to the dental structures, the highest stress value was registered at the buccal surfaces of the premolars and second molars, which were adjacent to force application sites. Clinical trials revealed successful results by the aid of mini-plates placed at the zygomatic buttress region for the intrusion of the maxillary posterior segment [4, 10, 11]. The intrusion appliance can be designed with acrylic bite blocks with heavy palatal arches to achieve block intrusion without buccal tipping of the posterior segment [4]. In this study, although we have constructed a similar design for the intrusion appliance, the stress distributions were not uniform for the anchorage teeth. Maximum stress values were observed at the buccal cusps of both premolars, as compared to palatal cusps, especially at the third scenario. In clinical situations, buccal tipping of the anchorage teeth can be observed during intrusion. Our result might indicate the increased tendency for buccal tipping, which might depend on the application of orthodontic force from the buccal site and also the relief effect of buccal and palatal corticotomies. In this respect, it is important to consider the morphology and surface area of roots, rigidity of the transpalatal arch, and the components of the orthodontic force. Çifter et al. [23] declared the importance of applying intrusive force both from buccal and palatal sites, in order to achieve a balanced stress distribution. Besides, it has been reported that force application from counterbalancing sites lead to a more balanced intrusion than using transpalatal arch, and that root surface areas of the anchor tooth has to be considered [23]. Similarly, force applications from the buccal and palatal sides, and using a transpalatal arch with sufficient resistance could be advised in order to prevent buccal tipping during posterior intrusion [37].

The apical third of the first molar mesiobuccal site showed the maximum stress, and the values were at least two times higher, when compared to other root apices. Again, apical region of the second molar demonstrated high stress values. Previously, Çifter et al. [23] found that the mesiobuccal root region of the first molar and first premolar presented the highest stress values under 300 g of force application. They related this finding to the small surface areas and geometrical structures of the related teeth. Li et al. [13] evaluated volumetric measurement of root resorption after molar intrusion using cone beam computed tomography, and their results showed that the highest volume loss was observed for the mesiobuccal root of first molar. The stresses at the apical region of posterior teeth produced by intrusive forces have a clinical relevance, since these areas can be prone to root resorption during posterior intrusion treatment [23, 38, 39].

When the stress values for root apices were considered in this study, no apparent change could be found related to the corticotomy simulations. Therefore, one may conclude that corticotomy application did not have an effect on the initial stresses acting on the apical regions. The stresses for the palatal apical regions were lower than that for the buccal apical regions, which might depend on the differences of the distance to force application point, variations of root angulation, and inclinations or diversities of bone morphology.

Taken together, as with any theoretical model of a biological system, evaluations through the finite element method reveal some limitations. First of all, the mechanical properties of the materials were assumed to be homogeneous and isotropic, different than in real clinical conditions, which should be interpreted with care. The patient’s age, bone thickness and quality, and the complexity of the disorder may affect the outcomes in clinical situations. Most importantly, only simulated stress distributions under initial forces along the supporting structures could be interpreted by finite element analysis. Therefore, clinicians should consider that the stresses along the supporting tissues can be changed because of the ongoing tooth movements and related bone remodeling responses, as well as the changes in the force systems.

## Conclusions

1. Corticotomies can affect the mechanical responses of dentoalveolar structures. Application of subapical corticotomies provided reduction of initial stresses along the cortical bone. Besides, increased stress distribution was found for cancellous bone around corticotomy regions, mostly on the buccal surface, which may provide accelerated bone turnover.
2. The apical third of the first molar mesiobuccal root showed the highest stress value, which might declare a tendency for root resorption. Due to the lack of an apparent difference at the stress values for root apices between models, intrusion could be performed without assistance of corticotomy or when needed, only a buccal corticotomy might be preferred.

## Abbreviations

3D: Three-dimensional
CT: Computed tomography
MPa: Megapascal
